# Gap study on concrete filled steel tube (CFST) and concrete filled double steel tube (CFDST) columns subjected to blast loads

**DOI:** 10.1038/s41598-025-27054-1

**Published:** 2025-12-05

**Authors:** M. Galal El Sherbiny, Mohammed H. Serror, Osman M. O. Ramadan, Ahmed M. Khalil

**Affiliations:** 1 Structural Engineering and Construction Management Department, Faculty of Engineering & Technology, Future University in Egypt - FUE, Cairo, 16383 Egypt; 2 Faculty of Engineering, Cairo University, Cairo, 12411 Egypt

**Keywords:** Blast loads, CFST column, CFDST column, Nonlinear analysis, Explicit dynamic analysis, Engineering, Materials science

## Abstract

Columns are essential structural components that must be assessed for potential hazards, including blast loads. Such loads pose significant risks to both civil and military infrastructure, whether caused by terrorist attacks or accidental explosions. This study conducts a comprehensive review of existing research on the blast response of concrete-filled steel tube (CFST) columns and concrete-filled double steel tube (CFDST) columns. Moreover, it includes recent research on the behavior of RC columns under blast loading, as they are a traditional and common structural shape. Furthermore, it reviews papers that discuss different methods for strengthening and retrofitting RC columns. The presented research focused on the performance of columns under close-in and contact blast loads, as these are considered more damaging and influential for columns than far-range blasting. Previous studies have examined the behavior of these columns using analytical, numerical, and experimental methods. The combined use of experimental and numerical approaches allows for a more thorough understanding of their dynamic response to blast loads. Furthermore, the study identifies key research gaps and suggests directions for future investigations in this field.

## Introduction

In recent years, the increasing frequency of terrorist attacks has elevated concerns about progressive collapse in bridges and buildings. Given that columns and bridge piers play a crucial role in transferring loads to foundations, extensive research has focused on mitigating their progressive collapse. This section examines blast phenomena, current design codes for blast-resistant structures, analytical methods for evaluating column response under blast loading, and typical damage modes and failure mechanisms in columns.

### Theoretical background

Explosions generate significantly higher strain rates (ranging from 100 to 10,000 s⁻^1^) compared to impact or earthquake loads^1^. An explosion involves the rapid and sudden release of energy, producing a blast wave with extremely high pressure, fragments from either the explosive material or surrounding structures, and hot gases reaching temperatures of approximately 3000–4000 °C and pressures of 100–300 kilobar. The blast wave propagates outward from the detonation source as a spherical wave. As the spherical radius expands over time, the surface pressure decreases. The peak pressure depends on the explosive quantity, material properties, and the distance from the explosion source to the structure^2^. Blast loading can be categorized into two main types: unconfined and confined explosions. Unconfined explosions are further classified into three subtypes: free-air bursts, air bursts, and surface bursts. Similarly, confined explosions are divided into three categories: fully vented, partially confined, and fully confined.

**Fig. 1:**
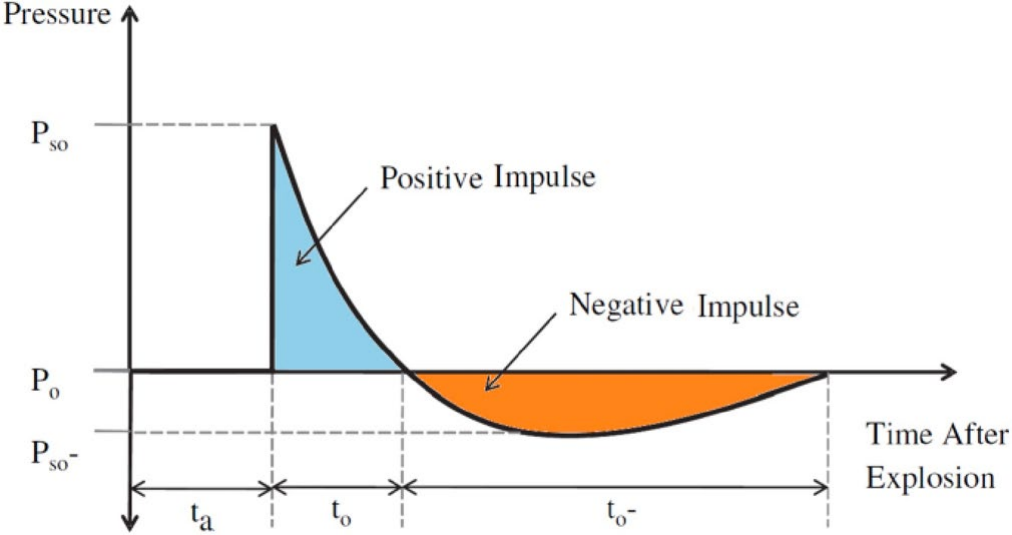
A typical plot of free-field blast pressure–time history^3^.

Figure 1 provides a complete illustration of a free-air burst. When detonation begins, the pressure rises from atmospheric pressure (P₀) to peak overpressure (Pₛₒ) during the positive phase, which has a duration of t₀. Subsequently, the pressure drops below P₀, reaching a negative pressure (Pₛₒ⁻) during the negative phase, which lasts for a duration of t₀⁻. The most common criterion for classifying blast loadings is based on the Hopkinson–Cranz law, which defines the scaled distance (Z) of detonation from the target. This parameter establishes a relationship between the equivalent weight of the explosive charge (W) and the standoff distance (R), expressed as follows:

1 $$Z = \frac{R}{{W^{1/3} }}$$

**Fig. 2:**
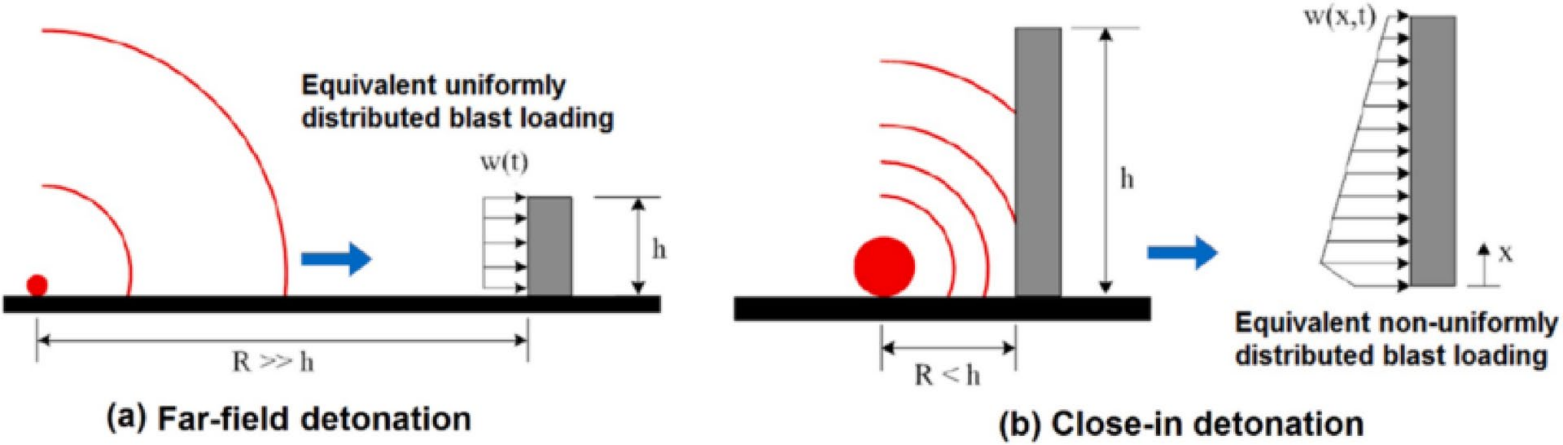
Reflected pressures on the target structures regarding the scaled distance of blast loading^3^.

Blast loads can be categorized based on the scaled distance parameter into near-field (close-in) and far-field detonations, as shown in Fig. 2. Close-in explosions are defined as those with a scaled distance (Z) less than 1.2 m/kg^1^/^3^, according to ASCE/SEI 59-11 (American Society of Civil Engineers). For far-field detonations, the blast pressure can be reasonably approximated as a uniformly distributed load. In contrast, close-in explosions produce more concentrated loading patterns, with pressure distribution becoming increasingly localized around the effective blast area as the scaled distance decreases^4,5^.

### Design codes for structures against blast loads and safety standards

Numerous guidelines exist for designing blast-resistant buildings, including those issued by the U.S. Department of the Army, U.S. Department of Defense, U.S. General Services Administration (GSA), Federal Emergency Management Agency (FEMA), and the American Society of Civil Engineers (ASCE). Additionally, the National Cooperative Highway Research Program (NCHRP) provides specific guidelines for protecting critical bridges against terrorist attacks. Table 1 summarizes the relevant design codes and guidelines for blast-resistant structural design^6–21^. Moreover, there are safety standards for non-structural elements and glazing mitigation as ISO 16933, ISO 16934, and AAMA 510.

**Table 1:** Summary of the current guidelines for blast loading on structures^3^.

Guideline	Remarks and notes
TM-5-855-1^17^	Provides design and analysis procedures for the protective structures exposed to the effects of conventional weapons and tor use in designing hardened facilities
TM-5-1300^18^	Classic US Army technical manual that established much of the blast design approach still in use (often referenced for legacy work and historical data). Provides design approaches for structures to resist the effects of blast waves and fragments by considering blast load parameters and structural response modes
o UFC 3-340-02^20^	o Used for the design of structures subjected to accidental explosions and used for antiterrorism design. Contains extensive empirical data, charts, and simplified methods (SDOF) analysis. It provides detailed methodologies for calculating blast loads and dynamic structural response. Prediction of idealized close-in and far-field blast loads using shock and gas considering dynamic increase factors (DIFs) which provide both flexural and shear
UFC 4-010-01^21^	Provides minimum antiterrorism standards for all depart of defense and military buildings. Focuses on layout, standoff distances, building hardening to mitigate terrorist threats. It defines levels of protection (LOP) from very low to high and provides required standoff distances or construction standards
FEMA 426^11^	Reference manual to mitigate potential terrorist attacks against buildings. Provide practical guidance to reduce vulnerability of new and existing buildings to terrorist threats. It content general site planning, security measures (access, standoff, surveillance), and overview of threats (blast, chemical, biological, radiological)
FEMA 427^12^	Provides primer for design of commercial buildings to mitigate terrorist attacks. It content structural system selection, progressive collapse mitigation, glazing/façade safety, building systems protection
FEMA 428^13^	Provides primer to design safe school projects in case of terrorist attacks. It content site access control, safe zones, evacuation planning, structural protection for children, balancing security with learning environment
FEMA 452^14^	Provides risk assessment to mitigate potential terrorist attacks. It content threat identification, vulnerability assessment, consequence analysis, risk rating tools/checklists
ASCE 1997^6^	Provides a structural design guideline for blast resistant of petrochemical facilities which often deal with accidental internal explosions rather than external terrorist threats. It provides practical guidance on design, detailing, and siting
ASCE/SEI 59-11^8^	Provide a modern, civilian engineering framework for designing and retrofitting buildings to protect life and property from blast effects, filling the gap between military-only standards and general building codes. Considers dynamic increase factors for structures for only far-range blast loads using single-degree-of-freedom (SDOF) analysis which provide flexural failure- based design approaches
ASCE (7-10)^7^	Provides the concepts and analysis methods of progressive collapse of integrated and redundant structural systems under explosions
NCHRP 12-72^15^	Provides effective methods, structural design, and retrofit guidelines to mitigate the risk of terrorist attacks against critical bridges
NFPA 59A^16^	Safety standards for Petrochemical facilities exposed to accidental vapor cloud explosions and blast loads. It defines siting, layout, and structural separation for worker and public safety

### Methods for analysis of structures under blast loading

**Fig. 3:**
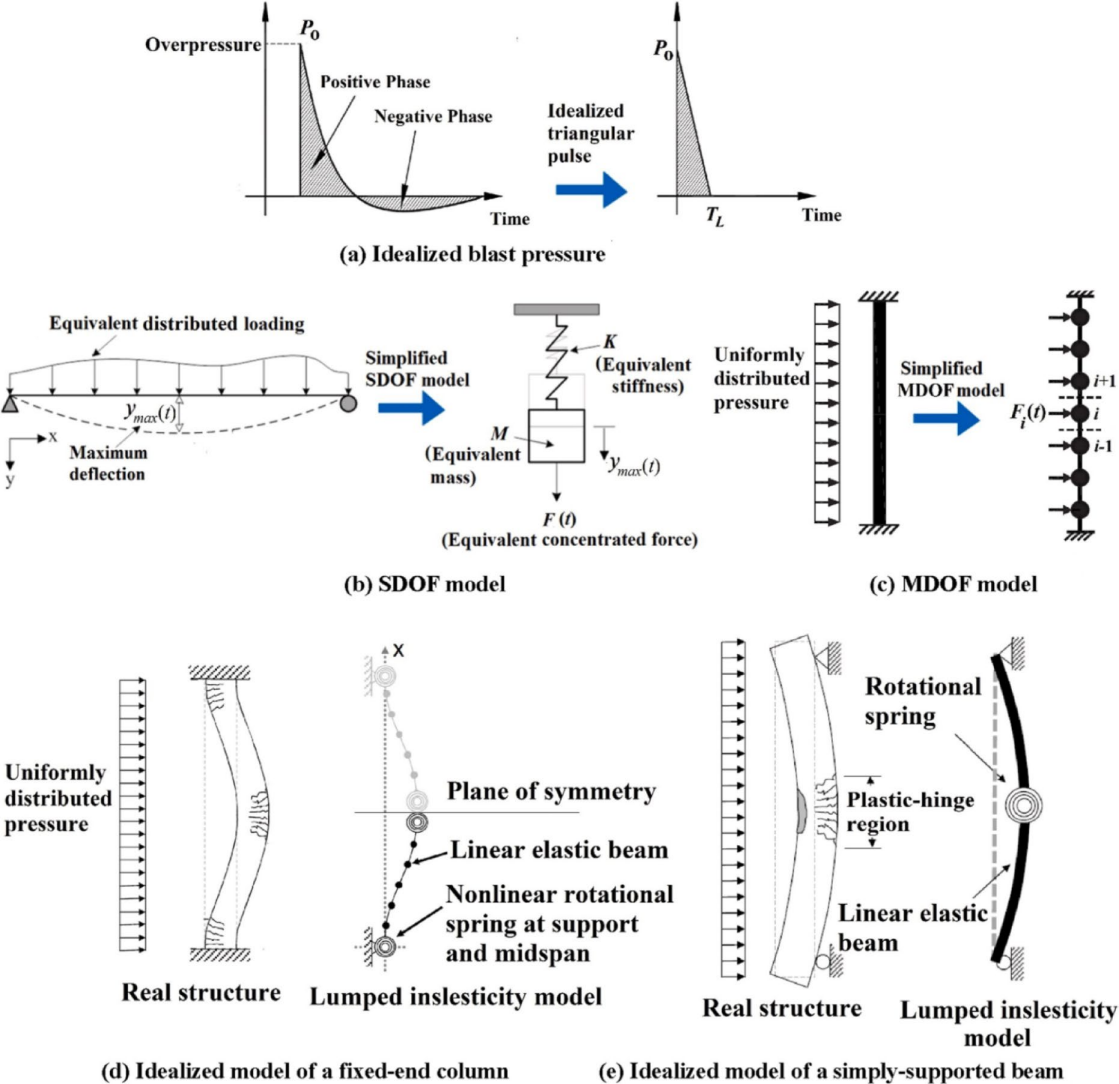
Examples of simplified models adopted by different analytical studies^3^.

There are three primary methods for studying structural behavior under blast loads: simplified analytical methods, numerical simulations, and experimental tests. Table 2 shows comparison between thee three methods in term of strengthen, limitations, and relevance. Many researches discussed blast load effects on structures through simplifications of both reflected pressure and structural systems^22^. As shown in Fig. 3a–e, analytical models often employ equivalent single-degree-of-freedom (SDOF) or multi-degree-of-freedom (MDOF) systems subjected to idealized blast loads. The derivation of resistance functions and load-transformation factors for these simplified models typically relies on idealized continuous systems or lumped inelasticity models (Fig. 3d–e). Several commercial software packages are commonly used for numerical analysis, including ABAQUS^23^, LS-DYNA^24^, and AUTODYN^25^. Four main experimental testing methods are employed in blast research: (1) Air burst tests using explosive charges, (2) Shock tube tests, (3) Tests using the University of California, San Diego (UCSD) blast simulator, and (4) Gas blast simulator (GBS) tests.

**Table 2:** Comparison between analytical, numerical, and experimental method to analysis effect of blast loads on structures.

	**Analytical**	**Numerical**	**Experimental**
Strengths	Provides rapid calculation for structure global behaviour and key design parameters	Provide more reliable results than analytical method Provides high accuracy to capture RC localized failures (spalling, scabbing, rebar fracture), also capture interaction between blast wave and structure It allows to easy study various design parametric such as (geometry, material, load, support, and soil-structure interaction)	It is the highest realism method to capture structure response and modes of failure It is best method to calibrating and validating analytical and numerical models
Limitations	Used for simple structural not complex one Use idealized load (uniform pressure) Cannot capture localized shear failures modes	Requires high computational cost Results accuracy depends on input parameters and requires experimental test to validate the numerical model	It is most expensive method due to it requires high quality equipment and Instrumentation to capturing the data from the experiment Requires safety and environmental constraints It is difficult to repeat the tests
Relevance	preliminary design for performance assessment of structural components against global failure	Used in analysis and design complex structures	Used in capture real structure behaviour and calibrate and validate the numerical model

### Damage modes of RC columns under blast loads

**Fig. 4:**
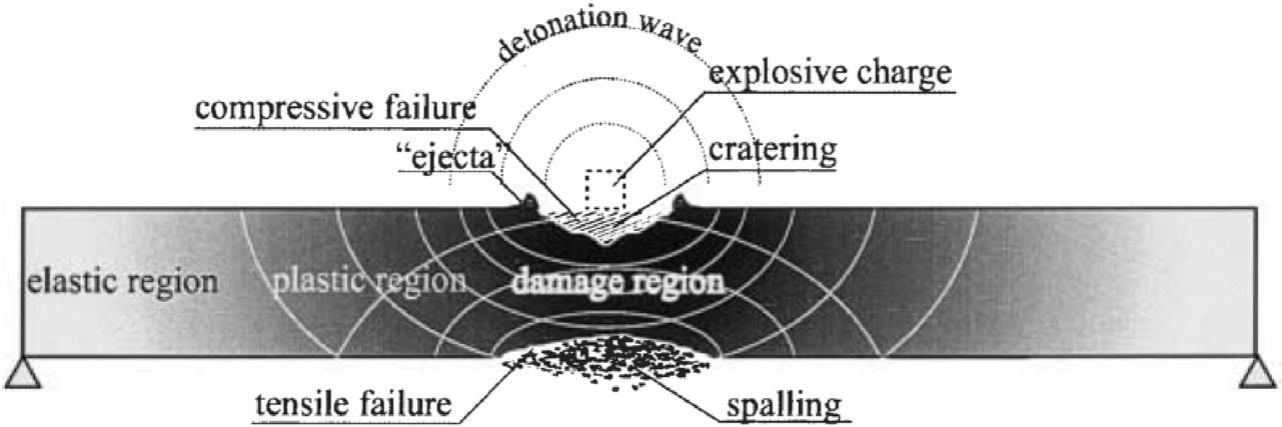
Localized failures in an RC structure under contact detonation^3^.

**Fig. 5:**
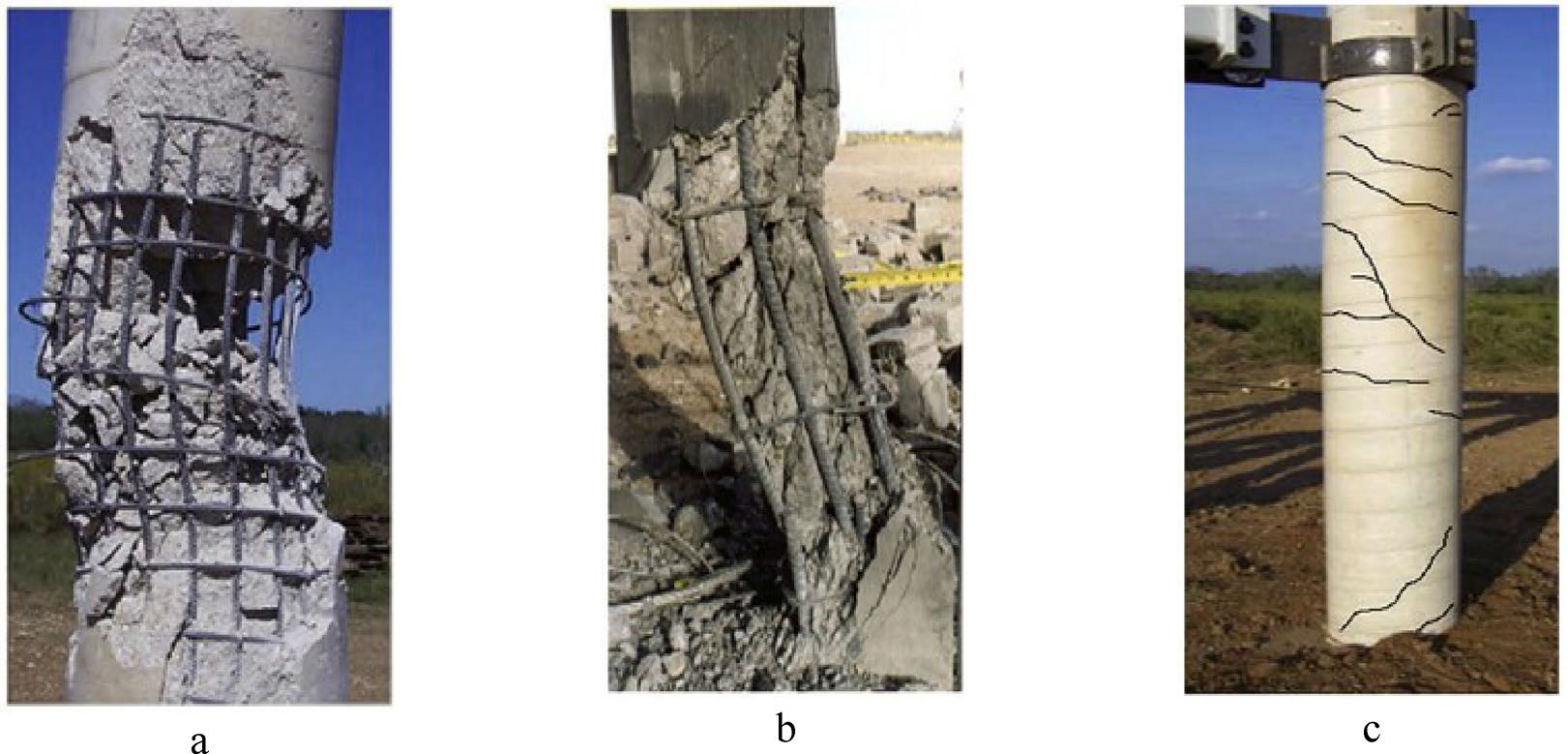
Typical failure behaviors of different RC columns under different blast loads: (**a**) Severe spallation, (**b**) Combination of localized and global failure, and (**c**) Global flexural damages^34^.

The damage modes of RC columns under blast loading can be classified according to scaled distance^26–29^. As shown in Fig. 4, contact and very close-in explosions cause varying severities of localized concrete spalling on the back surface due to tensile stresses induced by the suction force of the reflected blast wave^30,31^. Table 3 presents different intensities of spall damage in concrete resulting from reflected stress waves. For close-in explosions, columns typically experience a combination of global deformation and localized spall damage. In contrast, columns subjected to far-field explosions, under nearly uniformly distributed loads, exhibit global flexural and tensile damage due to their ductile behavior. Table 4 classifies the flexural failure modes of RC columns based on displacement-ductility ratios. The ductility ratio depends on material properties, member geometry, steel reinforcement and axial Load Fig. 5 illustrates three distinct damage modes for blast-loaded RC columns: (a) Localized concrete spalling under contact or very close-in explosions^32^, (b) Combined localized and global failure under close-in explosions^33^, and (c) Global flexural damage under far-field explosions.

**Table 3:** Spalling damage classifications^3^.

Damage state	Damage description	Scheme of damage
No damage	From no change in the conditionof the wall to a few barely visible cracks	
Threshold spall	From a few cracks and a hollow sound to a large bulge in the concrete with a few small pieces on the floor	
Medium spall	From a very shallow spall to a third of the wall thickness	
Severe spall	From just over one third the wall thickness to almost breach	
Breach	From small hole which barely lets light through to a large hole

**Table 4:** Flexural failure modes based on the displacement-ductility ratio^3^.

Failure mode	Damage description	Scheme of damage
Light flexure	From no permanent displacement but a few flexural cracks to a ductility ratio 3	
Medium flexure	From a ductility ratio of 3–10	
Severe flexure	From a ductility ratio of 10 to almost breach

## Response of RC columns to blasting

Kyei et al.^35^ conducted numerical simulations using LS-DYNA to investigate the influence of transverse reinforcement spacing on reinforced concrete (RC) column behavior under far-field blast loads. Their study examined multiple parameters, including explosive weight, standoff distance, and axial load ratio. The results demonstrated that decreased transverse reinforcement spacing reduced lateral displacement during close-in explosions, with the additional gravity load on the column further mitigating lateral displacement.

Momeni et al.^36^ utilized LS-DYNA to numerically assess the performance of steel columns with identical section properties under two different blast scenarios, each with varying explosive weights and standoff distances. Their analysis evaluated column damage, lateral displacement, and residual axial capacity, while also comparing the accuracy of different modeling approaches (beam, shell, and solid elements). For pinned-end columns subjected to blast loading, the beam, shell, and solid models produced similar maximum displacement predictions, with the shell and beam models showing less than 5% deviation from the solid model. However, for fixed-end columns, this deviation increased significantly to 30–35%.

Given the substantial costs of experimental blast testing, numerous researchers have adopted combined experimental–numerical approaches to study column blast response^37–41^. While studies^37,38,40,41^employed Autodyn for numerical modeling, Dua et al.^39^ used LS-DYNA. Codina et al.^37,38^ performed full-scale tests on RC members subjected to near-field explosions, comparing two parameter sets for the RHT concrete model: Autodyn’s default values and a modified set proposed by the authors. Their findings confirmed that the authors’ parameters effectively simulated RC column response at small scaled distances (0.3–0.5 m/kg1/3).

Dua et al.^39^ evaluated the response of large-scale square and rectangular concrete columns with varying aspect ratios under contact explosions using different explosive charges. Through experimental testing, the authors assessed the residual axial capacity of damaged specimens, finding that increasing the width of rectangular columns by 67% during contact explosions with 500 g TNT reduced the damage index from 1.0 to 0.71. Additionally, rectangular columns demonstrated lower damage indices than comparable square columns (with equivalent concrete volume and reinforcement) when subjected to contact explosions with 2.5 g TNT.

Hu et al.^40^ examined RC column responses to close-range explosions using double-end initiated explosive cylinders. Their study focused on the effects of charge shape, diameter-to-length ratio, scaled distance, and detonation point by measuring overpressure and reflected pressure. Results indicated that charge shape significantly affects peak reflected overpressure, with cylindrical charges producing twice the overpressure of spherical charges. Double-end initiation generated 2–3.5 times greater overpressure than center-point initiation.

Liu et al.^42^ conducted complementary experimental and analytical studies on half-scale RC beams and columns exposed to varying charge weights at different standoff distances. Their validated numerical model showed that RC beams experienced flexural failure, with reduced standoff distances causing progressively severe concrete damage—from minor surface cracking to bottom surface spallation. Both studies^12,43^ concluded that increased charge weight exacerbates concrete damage at constant standoff distances.

Li et al.^44^ experimentally evaluated residual axial capacity and damage in ultra-high-performance concrete (UHPC) and high-strength reinforced concrete (HSRC) columns subjected to different explosive charges at identical standoff distances. Their results demonstrated that microfiber-reinforced UHPC columns retained over 70% of load capacity after exposure to 35 kg TNT at 1.5 m, whereas HSRC columns maintained only 40% capacity following an 8 kg TNT detonation at the same distance.

Numerical simulations provide a crucial methodology for analyzing structural blast responses. Both structural component and surrounding air mesh sizes significantly affect result accuracy, particularly for contact explosions where air mesh sensitivity becomes critical^45^. Alok et al.^46^ performed numerical and experimental investigations of RC column responses to contact blasts, proposing an air mesh sensitivity analysis methodology that demonstrated substantial mesh size effects on pressure and impulse result accuracy.

Yang et al.^47^ and Zhuang et al.^48^ both investigated RC column responses to underwater explosions. Zhuang et al. conducted experimental studies examining circular column behavior under varying charge weights, standoff distances, and detonation depths. Yang et al. comparatively analyzed square versus circular column performance in both air and underwater contact/close-in blast scenarios, developing an Autodyn numerical model with RHT concrete material representation validated against experimental data. Zhuang et al. analyzed load distribution along column height, peak lateral displacements, and shock wave behavior (including reflection/diffraction effects), concluding that: (1) reflected wave pressures exceed diffracted pressures; (2) increased standoff distances reduce shock wave pressure; and (3) detonation depth significantly affects bubble pulsation pressure. Yang et al. found that cross-sectional shape critically influences dynamic blast response due to shock wave diffraction effects, recommending circular columns for superior blast resistance. Their work emphasized that underwater explosion shock wave diffraction effects—often negligible in air blasts—can cause substantial concrete damage.

L L et al.^49^ discussed analytical model and design approach of RC column/beam considering localized damage under near explosions and global response under far-range explosions. Firstly, they classify the local damage levels of 119 field explosion tests where they found that the critical scaled distance for local and global response is 0.78 m/kg^1^/^3^. Secondly, they developed an advanced (SDOF) model that considered non-uniform blast loading, strain rate effects, direct shear, flexural-shear, flexural responses P-Delta effects, and compressive arching effects. The model was validated by seven explosion tests with simply supported and fixed ends from aspect of direct shear, flexure shear, and flexure response. Ju et al.^50^ presented an improved analytical model for simulating the progressive collapse RC frame structures under blast loading. They focused on efficiently tracing collapse sequences in multi-story, multi-bay frames. They used beam elements instead of solid meshes to reduce computational cost. The parametric study included effect of bond-slip, catenary action, shear slip, and axial force on collapse behavior. They concluded that axial force significantly effect on collapse mode, while bond-slip, catenary, and shear effects were less effective global structural behavior. Yang et al.^51^ developed an analytical model to predict localized damage in reinforced concrete (RC) beams subjected to contact explosions. They Validated the model using experimental testing of four full scale Rc beams and numerical analysis in the study. The analytical model was developed to describe three damage stages initial impactor, explosion crater, and side damage. The parametric study included varied explosive weight, charge dimensions, and reinforcement ratio. They concluded that the proposed model predicted damage dimensions with an average error of 9% compared to experiments.

George et al.^52^ explored the fire-resistant behavior of rectangular (CFST) columns using steel fiber-reinforced concrete (SFRC). Fourteen columns with three concrete strength (M20, M30, M40), with and without steel fibers, were tested under ambient and elevated temperatures (1050 °C). Mechanical properties, axial load, ductility, and failure modes were evaluated. Results showed SFRC enhanced compressive, tensile, and flexural strength, with superior load capacity and ductility compared to conventional concrete. At high temperatures, performance declined due to fiber deterioration. Also George et al.^53^ discussed behaviour of light gauge steel hollow and concrete infilled structural subjected to temperature. Ruan et al.^54^ discussed numerically using ABAQUS the dynamic behaviour of (RC) columns subjected to combined fire and blast loads. The proposed numerical model was validated against experimental test. They examined different fire exposure scenarios on column faces (four-sided, three-sided, two opposite sides). Also they assessed the residual axial loading capacity and identified failure modes dependent on the fire scenario. The numerical model effectively predicted mid-span displacements, damage patterns, and residual strength of RC columns.

## Strengthen and retrofitting of Rc column

Some studies focused on assessed different method to strength and retrofit RC column to subject blasting^55–59^. Yan et al.^60^ investigated numerically and experimentally the blast resistance of RC columns strengthened with Carbon Fiber Reinforced Polymer (CFRP) sheets subjected to close-in explosions. The study showed the effect of CFRP thickness, bonding strength, anchorage systems, and axial load on column’s response. Twelve specimens of RC columns were tested experimental and numerical model by developed by LS-DYNA and validated with the experiment results. They concluded that CFRP significantly enhances blast resistance by reducing displacements and preventing spalling. Anas et al.^61^ numerical investigated blast resistance of axially loaded square RC columns subjected to close in blasting with different confinement strategies. They assessed effectiveness of single versus double layers of transverse steel confinement and the addition of Carbon-Fiber Reinforced Polymer (C-FRP) wrapping. The numerical model represented by ABAQUS and validated by replicating a past experimental test on a conventional column. The parametric study included number of steel confinement layers (single or double), the spacing of the transverse stirrups, the geometry of the inner confining stirrups, and use of 2 mm thick CFRP wrapping. They concluded that double confinement significantly reduces concrete crushing and cracking. CFRP wrapping drastically improved performance of column where it reduced displacement by up to 84% and damage energy by 95%.

Chen et al.^62^ discussed numerically the progressive collapse behavior of a twelve-story (RC) frame building under blasting and evaluates the effectiveness of two retrofitting measures: fiber-reinforced polymer (FRP) wraps and steel plate (SP) jackets. The parametric study included effect of scaled standoff distances (0.55 and 1.0 m/kg^1/3^) on Frame behavior. The results showed that SP retrofitting prevented collapse at both distances, while FRP was effective only at the larger distance. The study concluded that both retrofitting methods enhanced blast resistance, but SP jackets offer superior performance in near-range explosion scenarios. Li et al.^63^. discussed numerically the blast response of FRP-retrofitted RC columns by evaluating failure modes of these columns. The numerical model was produced by LS-DYNA and validated with experimental data from quasi-static, field blast, and shock tube tests. They concluded that FRP wrapping significantly enhanced shear capacity and ductility, changing the column’s failure mode from a catastrophic diagonal shear to a more ductile flexural failure.

## Response of CFST columns to blasting

Recent studies have increasingly focused on the blast behavior of concrete-filled steel tube (CFST) columns^34,64–67^. Table 5 shows summary of those studies. Numerical investigations of square CFST columns under close-range explosions were conducted by^64,65^, with Ritchie et al.^65^ additionally performing experimental tests and developing an analytical model using a single-degree-of-freedom (SDOF) approach. Both research teams^64,65^employed LS-DYNA to compare the performance of concrete-filled and hollow steel tube sections. Ngo et al.^64^ validated their numerical model against published experimental data for square CFST columns subjected to close-range explosions. Their study examined standoff distances of 100 mm and 150 mm using a constant 2.6 kg TNT charge. In contrast, Ritchie et al.^65^ investigated column response to larger explosive charges (500 kg and 1000 kg TNT) at standoff distances of 15 m and 27 m, respectively.

**Table 5:** Summary of the studies on CFST column under blasting.

Paper	Analysis type	Section geometry	Section dimensions(mm)	Type of blast	Parametric study
Ngo et al.^72^	Nu	Square	100 × 5	Close	Concrete filled effect Standoff distance
Ritchie et al.^42^	Ex& Nu& An	Square	120 × 5 120 × 8	Far	Concrete filled effect Tube thickness Cross section dimensions Concrete strength Scaled distance
Zhang et al.^73^	Nu	Square & Circular	200 × 2.8 200 × 3.8 200 × 3.8 (Dxt)	Close	Explosive charge Axial load Cross-section Geometry
Wang et al.^69^	Ex	Square & Circular	200 × 2.8 200 × 3.8 194 × 2.8 (Dxt) 194 × 3.8 (Dxt)	Close	Cross-section geometry Charge weight Steel tube thickness Axial load
Wang et al.^74^	Ex	Square	100 × 1.5	Close	Recycled aggregate ratio Standoff distance Charge weight Steel tube thickness Explosion height
Wang et al.^65^	Ex	Circular	203 × 6	Contact	Charge weight
Peng et al.^63^	Ex & Nu	Rectangular	200 × 4	Contact	Recycled aggregate ratio column height Steel tube thickness Steel yield strength Charge weight

**Fig. 6:**
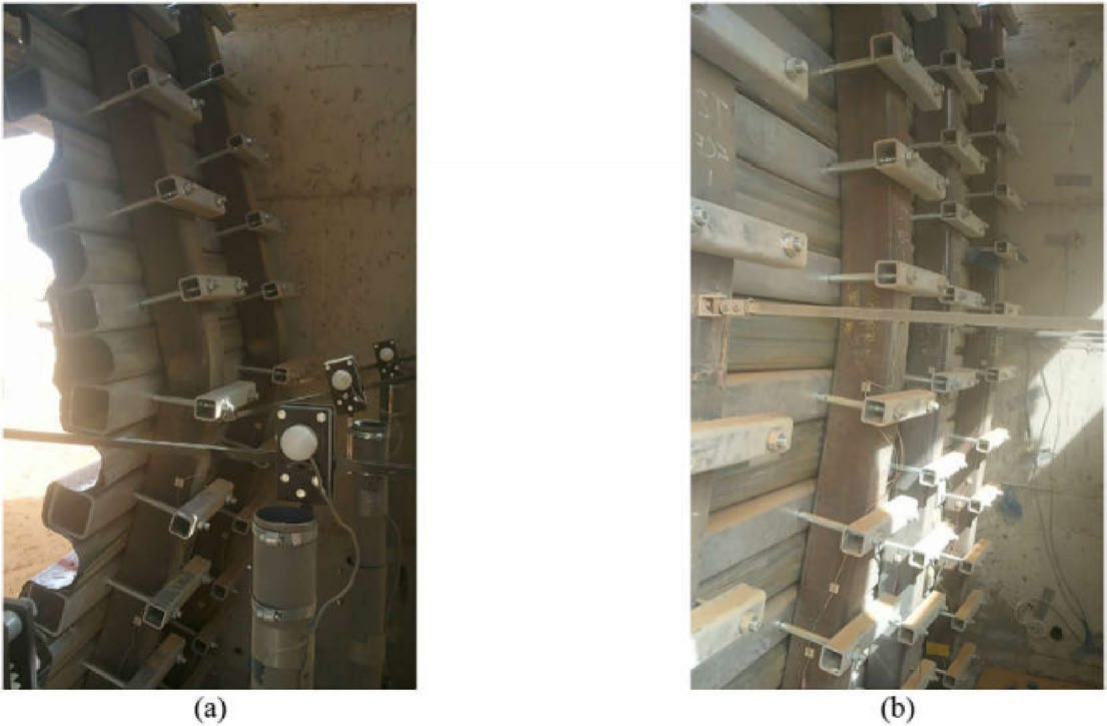
CFST after close in blasting: (**a**) unfilled concrete, (**b**) filled concrete^65^.

The research methodologies differed significantly: Ritchie et al. instrumented test columns (both filled and unfilled) with strain gauges to assess damage and deformation (Fig. 6), while Ngo et al. focused on analyzing pressure–time histories, displacement profiles, and strain development. Both studies identified two distinct deformation phases: (1) initial local tube deformation, followed by (2) global flexural deformation, with energy absorption occurring through both steel tube deformation and concrete crushing. A key finding from Ritchie et al.^65^ was that concrete infill substantially reduces both global and local deformations under blast loading. The researchers concluded that while the concrete’s primary role is to provide additional mass that prevents local buckling of the steel tube, its compressive strength also contributes significantly to the composite column’s blast resistance.

Two seminal studies^34,67^investigated the performance of square and circular concrete-filled steel tube (CFST) columns under near-range explosions. Wang et al.^34^ carried out experimental evaluations of CFST column blast resistance, whereas Zhang et al.^67^ performed LS-DYNA numerical simulations validated against experimental data, including three-point bending and field blast tests. Wang et al. tested eight large CFST columns 2500 mm in height: four square columns with 200 mm side lengths and four circular columns with 194 mm diameters. Their experimental program also included three-point bending tests to examine static behavior under combined lateral and axial loading. Wang et al.^34^ concluded that the failure modes of circular and square specimens were different. Square columns failed due to concrete crushing and spalling, whereas circular columns failed because the concrete filler split horizontally into three segments at the weak areas created by the blast loads. Moreover, square specimens exhibited larger residual axial load ratios than circular specimens under the same blast loads.

**Fig. 7:**
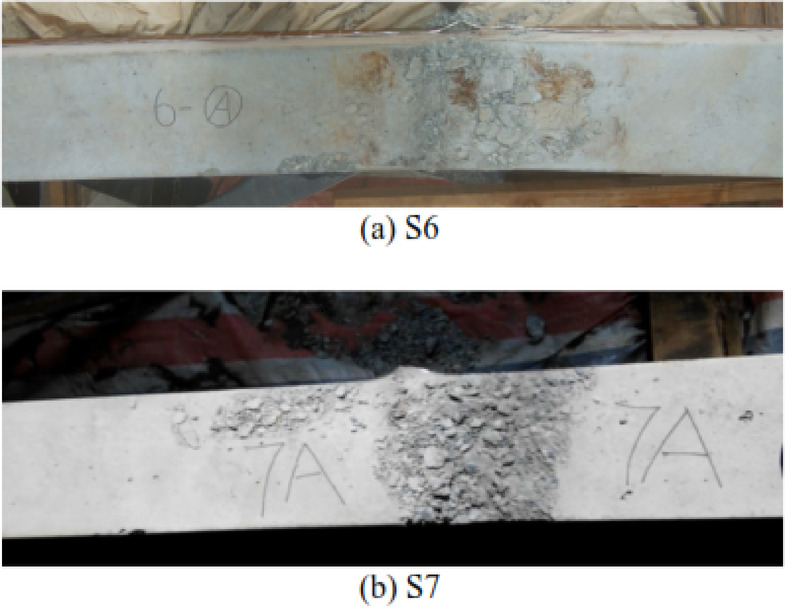
Concrete crushing and spalling of square columns^34^.

**Fig. 8:**
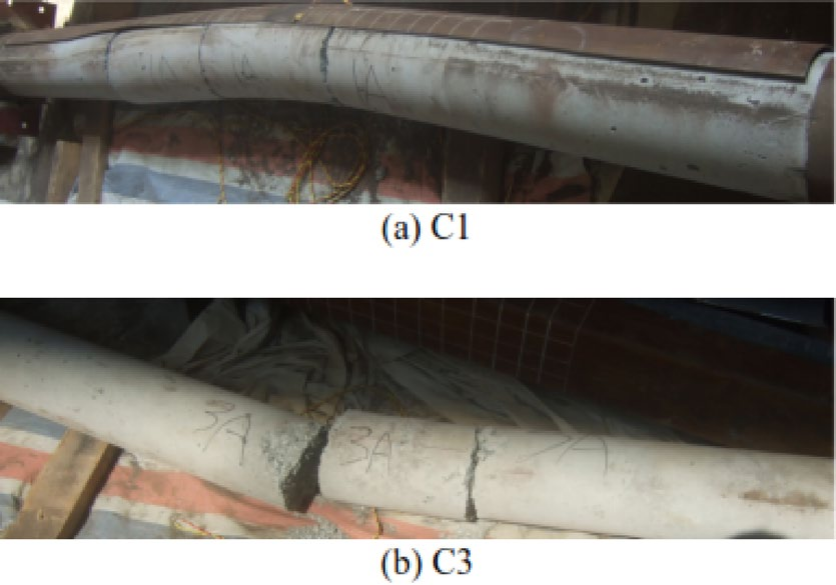
Concrete split of circular columns^34^.

Both studies^34,67^ considered axial load effects: Wang et al. focused on residual axial capacity and displacement–time histories, while Zhang et al. analyzed damage patterns, displacements, and pressure distributions. Zhang et al. concluded that CFST columns dissipate blast energy primarily through global deformation, with the concrete infill providing crucial protection against local steel tube deformation. Wang et al. conducted a comprehensive parametric study examining axial load magnitude, explosive charge weight, steel tube thickness, and cross-sectional geometry (square versus circular). Key findings included:

- Greater explosive charge weight produced larger mid-span displacements.
- Thicker steel tubes resulted in reduced displacements.
- Square columns failed through concrete crushing and spalling (Fig. 7).
- Circular columns failed through separation of the concrete filler into three horizontal segments (Fig. 8).

**Fig. 9:**
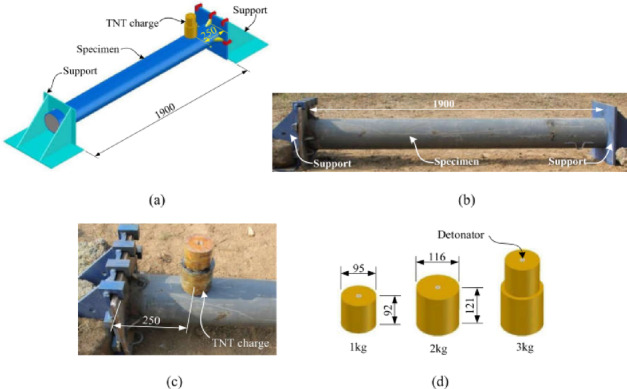
CFST column subjected to field contact explosion: (**a**) Schematic view, (**b**) photograph, (**c**) explosive layout, (**d**) explosive dimensions^66^.

**Fig. 10:**
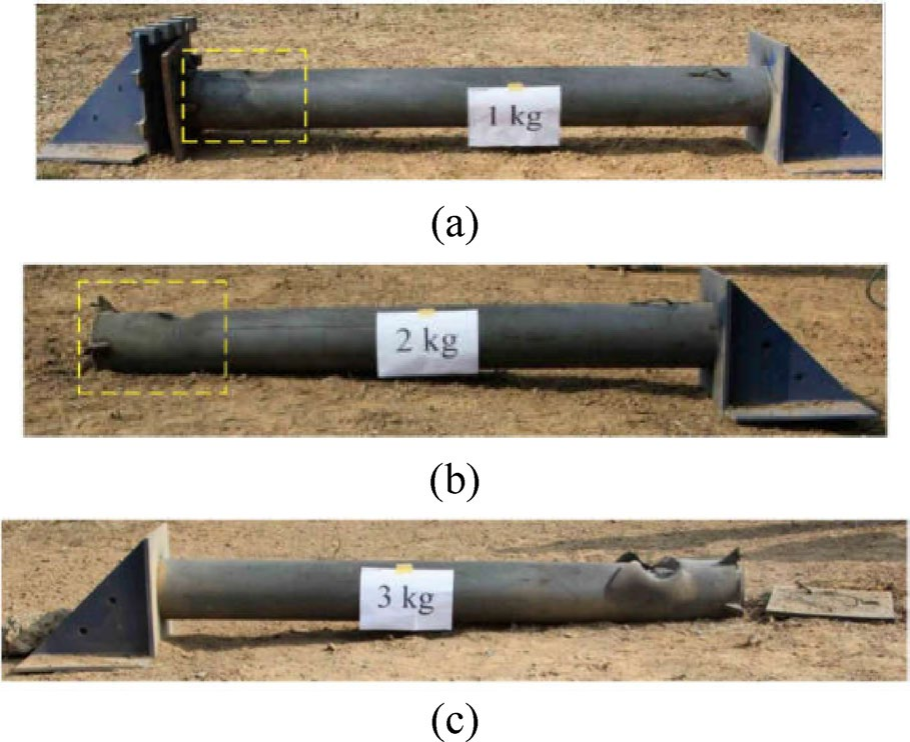
Post blasting CFST columns: (**a**) C1, (**b**) C2, (**c**) C3^66^.

Wang et al.^66^ conducted experimental investigations on ultra-high-performance cementitious composite (UHPCC)-filled steel tube columns using five 1/4-scale circular specimens. The columns measured 2000 mm in height and 203 mm in diameter, with 1900 mm between supports—featuring a fixed base and a pinned top support condition. Three columns were subjected to contact explosions with varying TNT charges of 1, 2, and 3 kg, as shown in Fig. 9, while the remaining two served as reference specimens for axial capacity evaluation. Wang et al. analyzed failure modes and damage patterns of the blast-exposed columns, as shown in Fig. 10, and assessed their residual axial capacity. Their findings revealed that:

• The first two columns effectively absorbed blast energy through the combined action of the steel tube and UHPCC core.

• The third column experienced rupture with complete UHPCC core crushing.

• A relationship was formulated between axial load and the corresponding axial and lateral displacements.

**Fig. 11:**
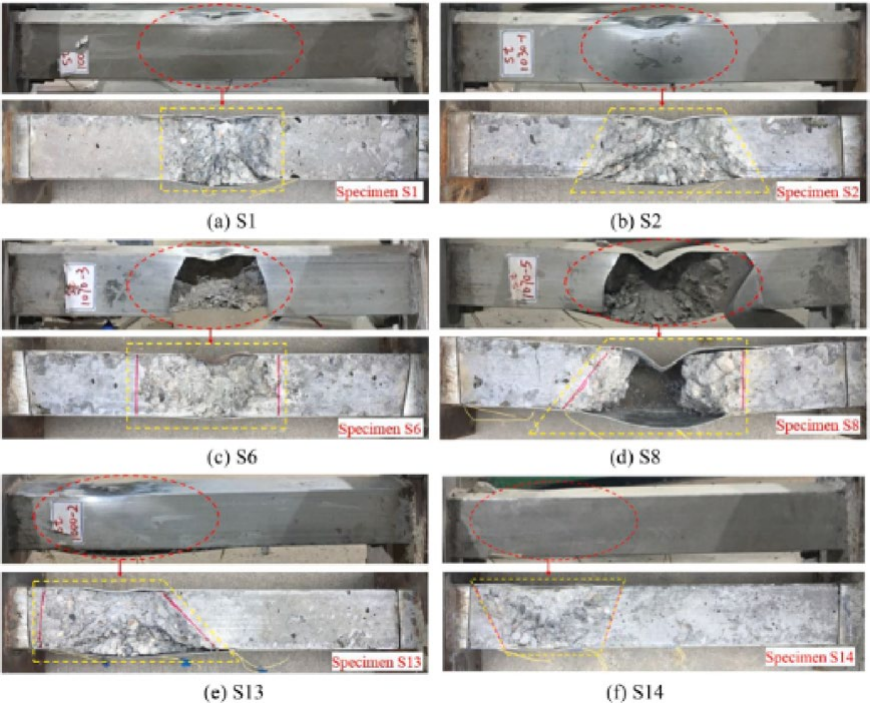
Failure of blast damaged columns after explosion test^68^.

Wang et al.^68^ investigated the residual axial capacity and damage of square recycled aggregate concrete-filled steel tube (RACFST) columns subjected to close-in explosions. The columns contained a mixture of natural aggregate and recycled aggregate from concrete members with over 30 years of service life. The study examined eighteen 1/5-scale square specimens (100 mm width × 660 mm height). Fifteen columns were exposed to explosions to evaluate various parameters, including replacement ratio, blast center distance, charge weight, steel tube thickness, and explosion height. The researchers analyzed failure modes and damage patterns of both the steel tubes and concrete cores (Fig. 11). Axial load tests were performed on nine blast-damaged columns and three intact reference specimens. Key findings included:

1. Both blast-damaged and intact columns exhibited shear failure under axial loading.
2. Steel tube thickness and core concrete strength significantly influenced blast resistance and residual capacity.
3. Columns with > 70% recycled aggregate (RA) replacement ratio sustained moderate damage, while those with < 70% RA showed only slight damage.

Peng et al^69^ conducted complementary numerical and experimental studies on rectangular RACFST (RRACFST) columns under contact explosions. Three experimental tests (validated through LS-DYNA simulations) evaluated failure modes, dynamic strain rates, and acceleration responses. The square columns (200 mm × 1500 mm) featured fixed-end supports, 40 MPa core concrete, and 4 mm steel tube thickness. Tests employed three charge weights (20 g, 30 g, 40 g TNT) detonated at mid-span. Their parametric study examined recycled aggregate replacement ratio, column height, steel tube thickness, and steel strength. They found that RRACFST columns suffered local damage at the detonation point, with the size of the explosive crater increasing as charge weight increased. Steel tube thickness was the dominant factor affecting column performance compared to the other factors.

Chen et al.^70^ developed a numerical model using LS-DYNA to evaluate the integrative damage of steel tubular columns subjected to blasting and post-explosion fire. The paper aimed to assess the reduction in mechanical and geometrical properties of a column’s fire resistance subjected to blasting. The parametric study investigated the effects of column height, width, and thickness. They found that blast damage significantly reduced the fire resistance; also they proposed P-I-t diagram can predict the residual capacity of columns under these combined threats. Ding et al.^71^ assessed numerically and experimentally the damage of Reactive Powder Concrete-Filled Steel Tube (RPC-FST) columns exposed to fire and subsequent blast loading. They evaluated the residual blast resistance of fire-damaged composite columns. The parametric study investigated the effects of damping ratio, ductility ratio, fire duration, and scaled blast distance. They concluded that columns with blast resistance decreasing significantly after fire exposure, and damage was more sensitive to fire duration than explosive charge weight.

## Response of CFDST columns to blasting

**Fig. 12:**
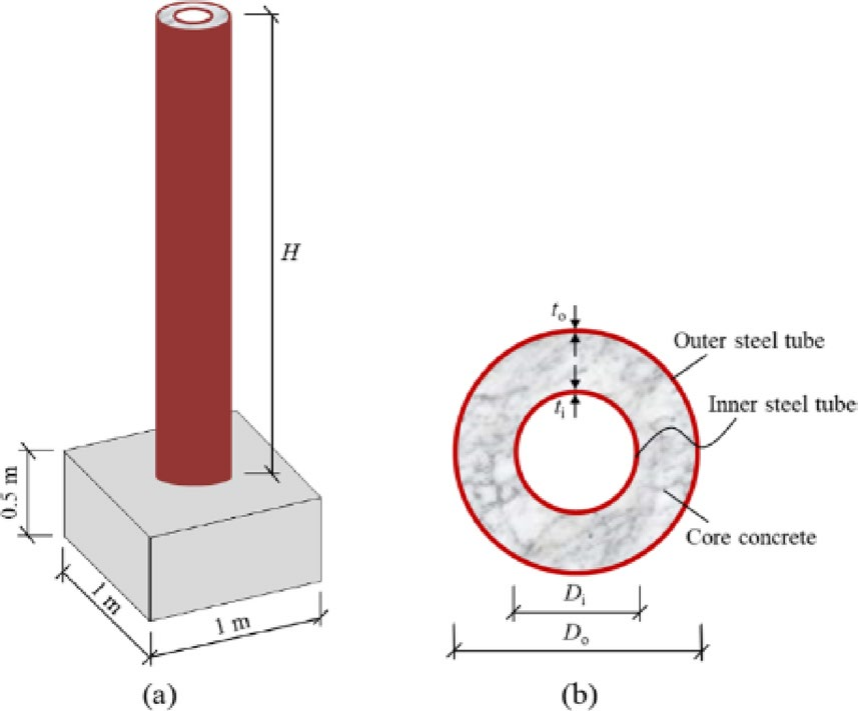
CFDST column specimen: (**a**) geometric configuration, (**b**) cross section of CFDST^75^.

**Fig. 13:**
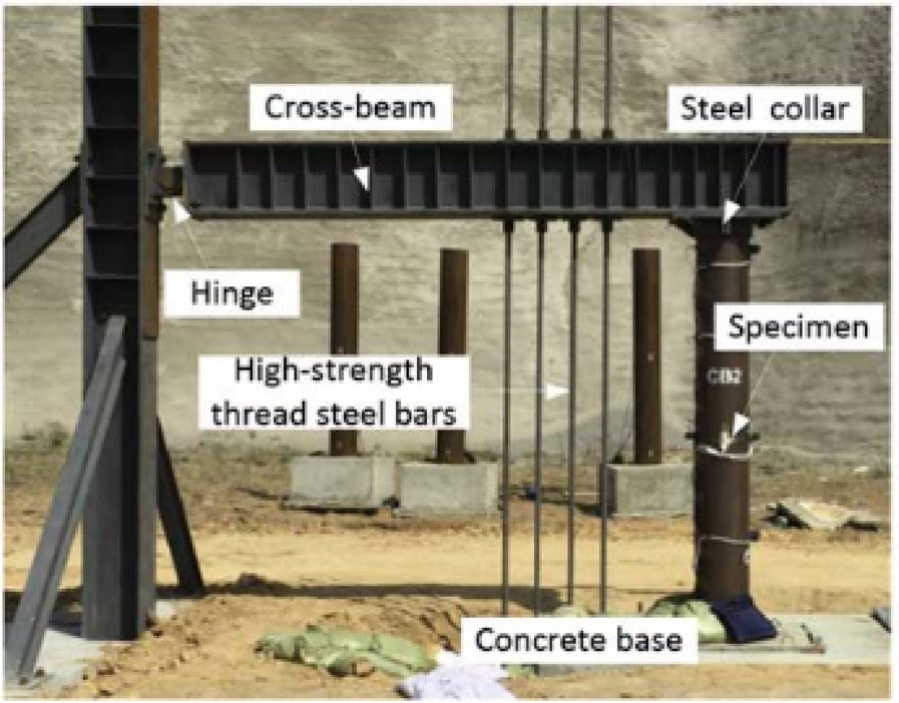
Field blast test setup^76^.

Some researchers have studied the response of concrete-filled double steel tube (CFDST) columns^44,72,75–81^ under contact and close-range explosions. Table 6shows summary of those studies. Li et al.^44,72,75–77^ explored the numerical and experimental performance of CFDST circular columns under contact and near-range explosions. Field tests utilized full-scale specimens, while LS-DYNA facilitated numerical simulations. The specimens were fixed in concrete blocks measuring 1 m × 1 m × 0.5 m (length × width × thickness), as shown in Fig. 12, while their tops were hinged, as shown in Fig. 13. The specimens measured 2.5 m in height, featuring an outer steel tube with a diameter of 325 mm and a thickness of 6 mm, alongside an inner tube measuring 159 mm in diameter and 6 mm in thickness. The axial load on the column is considered in^72,75^, while omitted in^44,76,77^.

**Table 6:** Summary of the studies on CFDST column under blasting.

Paper	Analysis Type	Section Geometry	Section Dimensions (Outer; Inner) (mm)	Type of Blast	Parametric Study
Li et al.^83^	Nu & Ex	Circular	325 × 6; 159 × 6	Close	Charge weight Standoff distance Axial load Charge orientation
Li et al.^84^	Nu & Ex	Circular	325 × 6; 159 × 6	Contact	Inner tube thickness Charge weight
Li et al.^40^	Nu & Ex	Circular	325 × 6; 159 × 6	Contact	Outer tube thickness Boundary conditions Axial load ratio Column height Cross-section dimensions Hollow ratio Nominal steel ratio Charge weight
Li et al.^85^	Nu	Circular	325 × 6; 159 × 6	Close	Charge weight Standoff distance Cross-section dimensions Nominal steel ratio Column height Axial load ratio
Li et al.^50^.	Ex	Circular	325 × 6; 159 × 6	Close & Contact	Charge weight Standoff distance Charge orientation Hollow ratio
Zhang et al.^68^.	Nu & Ex	Square	210 × 5; 110 × 5	Close	Axial load, Filler material-UHPC
Zhang et al.^66^.	Ex	Square & Circular	210 × 5; 110 × 5	Close	Cross-section geometry Tube thickness Hollow ratio
Zhang et al.^86^.	Ex	Square & Circular	210 × 5; 100 × 5	Close	Cross-section geometry Axial load
Zhang et al.^87^.	Nu	Square	210 × 5; 110 × 5	Close	Cross-section dimensions Column height
Xia et al.^17^.	Nu & Ex	Circular	325 × 6; 159 × 6	Close	Boundary conditions Friction between segments

**Fig. 14:**
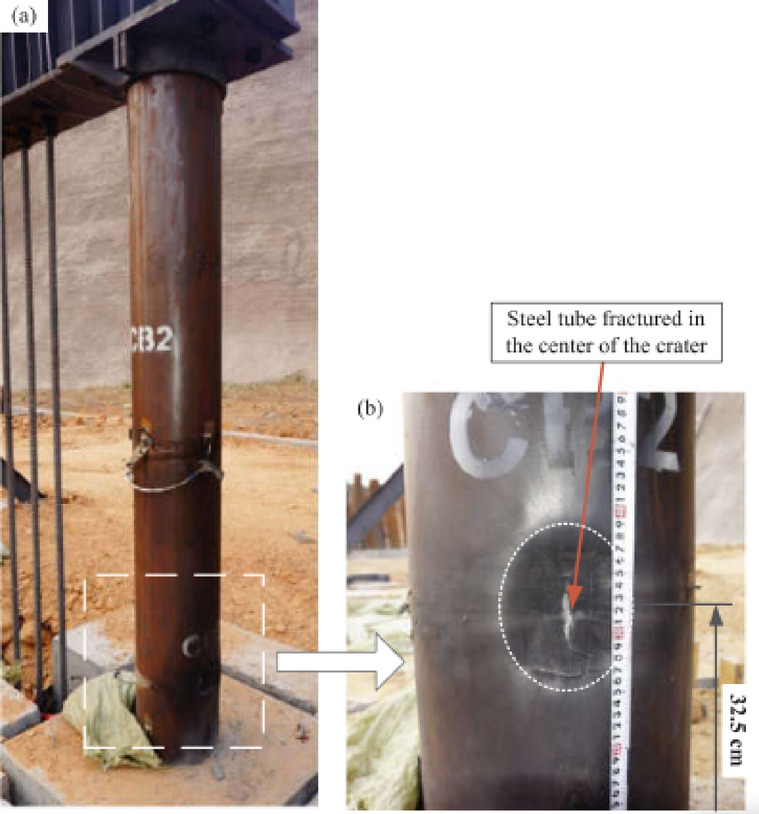
Field observation of specimen CB2 to contact explosion^44^..

**Fig. 15:**
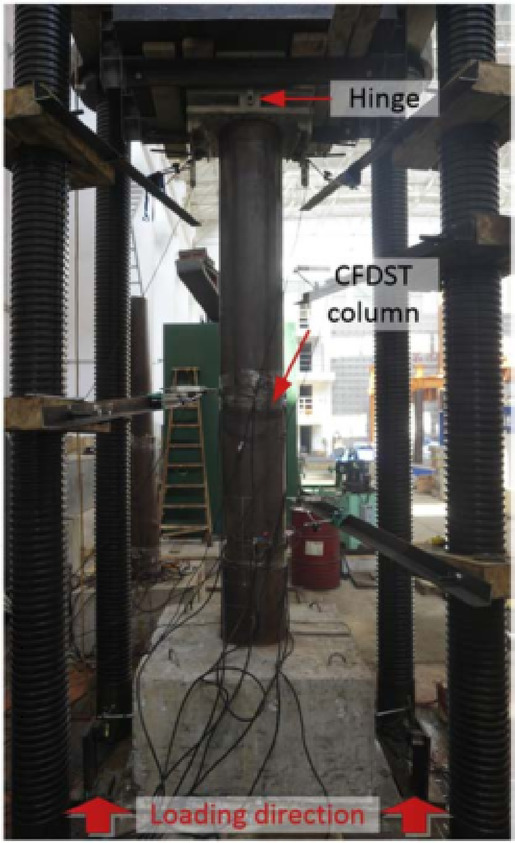
Axial compression test of post blast columns^76^.

Li et al.^75^ investigated, numerically and experimentally, the CFDST columns’ responses to close-in blast loads using two explosive charge weights at two standoff distances. Li et al.^44,76^ focused on the columns’ responses to contact explosions, numerically and experimentally, employing two different explosive charge weights. In^44^, they evaluated the deformed shape and damage mechanism of columns, as shown in Fig. 14, while in^76^, they assessed the residual axial capacity of post-blast columns, as shown in Fig. 15. Li et al.^72^ studied numerically the residual axial capacity of damaged CFDST columns subjected to near explosions, while Li et al.^77^ evaluated experimentally the damage mode and residual axial capacity of post-blast columns after near and contact explosions. Li et al.^75,77^ also investigated the effect of explosive charge orientation on column performance.

Study^44^ introduced several inner tube thickness variations, while study^76^ assessed variations in outer tube thickness. Researchers evaluated the damage and deformation of the tested columns, along with the energy of numerical models in studies^44,75^. Notably, study^76^ provided a post-blast assessment of the static axial capacity, both numerically and experimentally, incorporating a robust parametric analysis of factors such as boundary conditions, axial load, column height, outer tube diameter, hollow ratio, and nominal steel ratio. Additionally, the specific impact of column axial load on its blast performance was examined in^75^. Li et al.^72^ assessed several parameters through calculation of a damage index, including charge weight, standoff distance, cross-section diameter, nominal steel ratio, column height, and axial load ratio. Li et al.^77^ conducted an extensive parametric study of various factors such as charge weight, standoff distance, charge orientation, and hollow ratio. Li et al.^75,77^ found that the orientation of the explosive charge significantly affects the column’s blast performance. The columns displayed local denting at the cross-section facing the explosion, with this deformation serving as an energy dissipation mechanism. They concluded that axial load has a limited impact on the column’s structural response. Furthermore, Li et al.^44,75^ discovered that concrete absorbs blast energy, while the outer steel tube dissipates energy through deformation, thereby protecting the concrete core against spalling damage.

In study^76^, an empirical formula was developed to assess damage levels in CFDST columns, and it was identified that increasing both cross-sectional area and structural steel ratio enhances their blast resistance. It was also noted that boundary conditions exert minimal influence on structural response. Li et al.^72^ concluded that the residual axial capacity of CFDST columns under close-in explosions increases with cross-section diameter and steel ratio. Furthermore, they found that the presence of axial compression on the column, regardless of its percentage, increases the residual axial capacity. Li et al.^77^ concluded that charge weight and standoff distance significantly influence the blast response of columns. Moreover, they found that CFDST columns with larger hollow ratios exhibit greater cross-section denting deformation under near-blast loading, especially for ratios larger than 0.5.

**Fig. 16:**
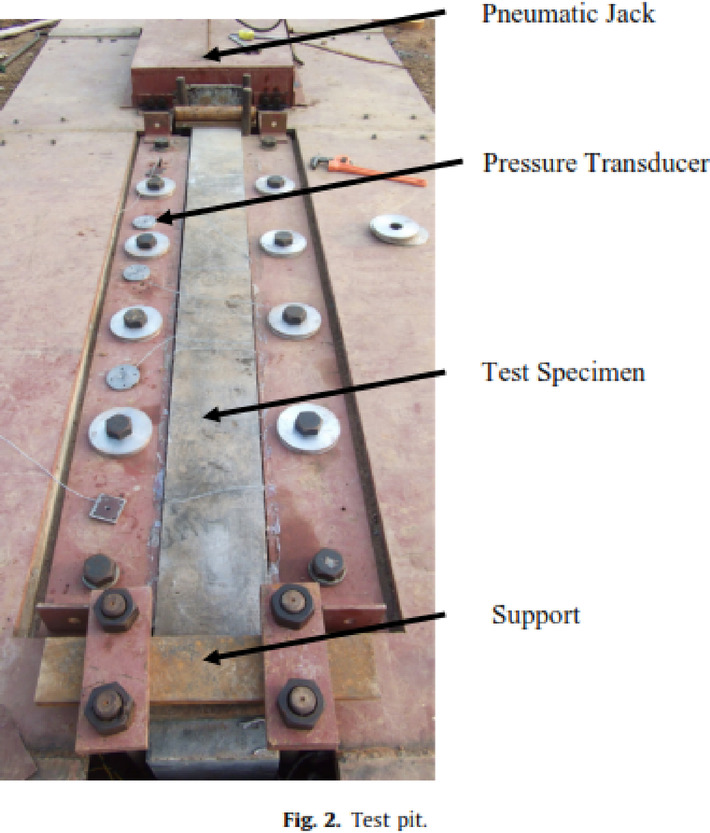
Test pit^78^.

**Fig. 17:**
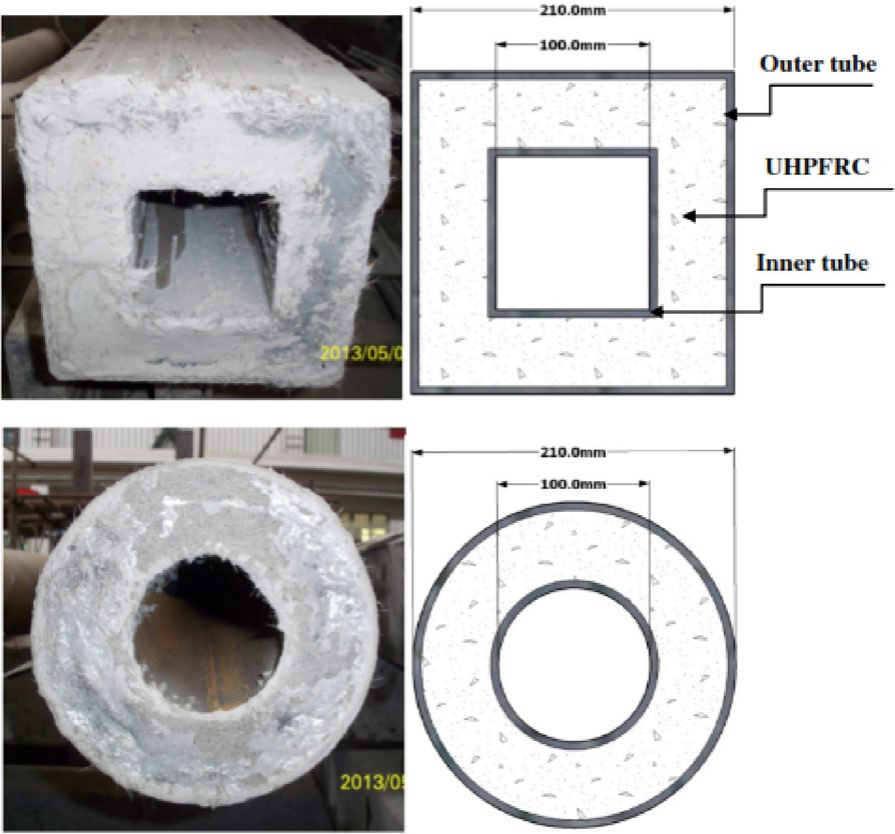
Cross section of a square and a circular CFDST specimen^79^.

Zhang et al.^78–81^ conducted experiments to evaluate the response of square and circular CFDST columns under close-range blast loading. In papers^79,80^, the case study included both square and circular CFDST columns, while in papers^78,81^, only square shapes were examined. The filler material inside the tubes was ultra-high-performance fiber-reinforced concrete. Zhang et al.^78^ utilized field tests with large-scale specimens, as shown in Fig. 16, and complemented their efforts with numerical simulations using LS-DYNA, while in paper^81^, they relied solely on numerical simulations validated against results from paper^78^. The case study remained consistent across the four papers, with columns measuring 2.5 m in height and an outer tube of 210 mm in diameter and 5 mm in thickness, alongside an inner tube measuring 110 mm in diameter and 5 mm in thickness, as shown in Fig. 17 except in^80^, where the inner tube was 100 mm in diameter and 5 mm in thickness.

The columns were subjected to various explosive charge weights at a constant standoff distance, with axial load considerations applied in both experimental and numerical analyses. Mid-span displacement was computed to assess the numerical models^78^. Damaged specimens were evaluated through comparisons of pressure and displacement^78,79^, and the residual axial capacity of the tested columns was calculated experimentally^80^. Diverse parameters, including axial load, hollow ratio, outer thickness, inner thickness, cross-sectional geometry, and explosive weight, were discussed across the four studies^78–81^.

Zhang et al.^78,79^ reported that utilizing a UHPC core reduces residual deflection compared to conventional-strength filler, while an increase in axial load ratio produces a marginal reduction in mid-span deflection. The studies indicated that increasing the thickness of either the outer or inner tube minimizes maximum deflection, with outer tube thickness having a more pronounced effect. The findings suggested that both CFDST and CFST columns exhibit comparable behaviors, with hollow ratios having a negligible influence on the overall column response^79^. Additionally, all damaged CFDST specimens maintained a damage index of less than 0.4, retaining over 60% of their axial load capacity following detonation^80^. Zhang et al.^81^ found that the key parameters influencing both pressure and impulse are cross-section dimensions and column height.

**Fig. 18:**
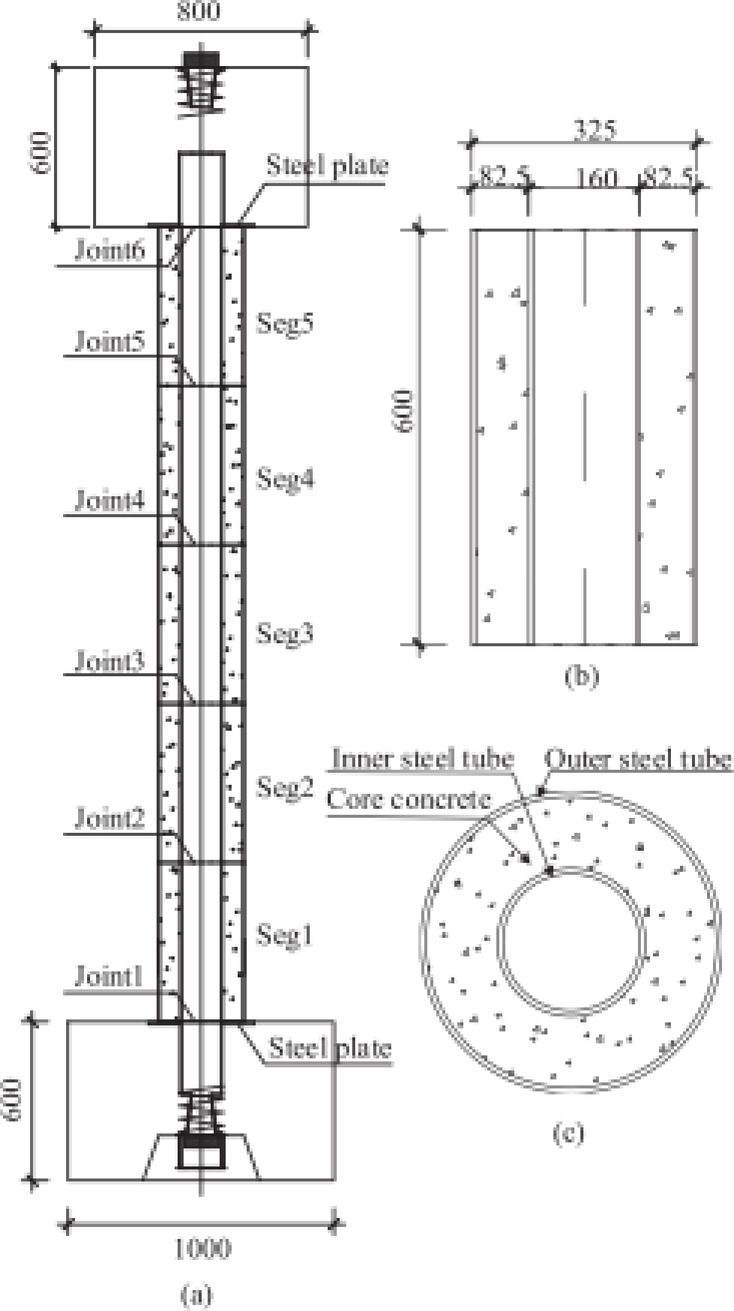
Schematic view of PS-CFDST specimens: (**a**) overall configuration, (**b**) details of CFDST segment, (**c**) cross section of CFDST segment^82^.

**Fig. 19:**
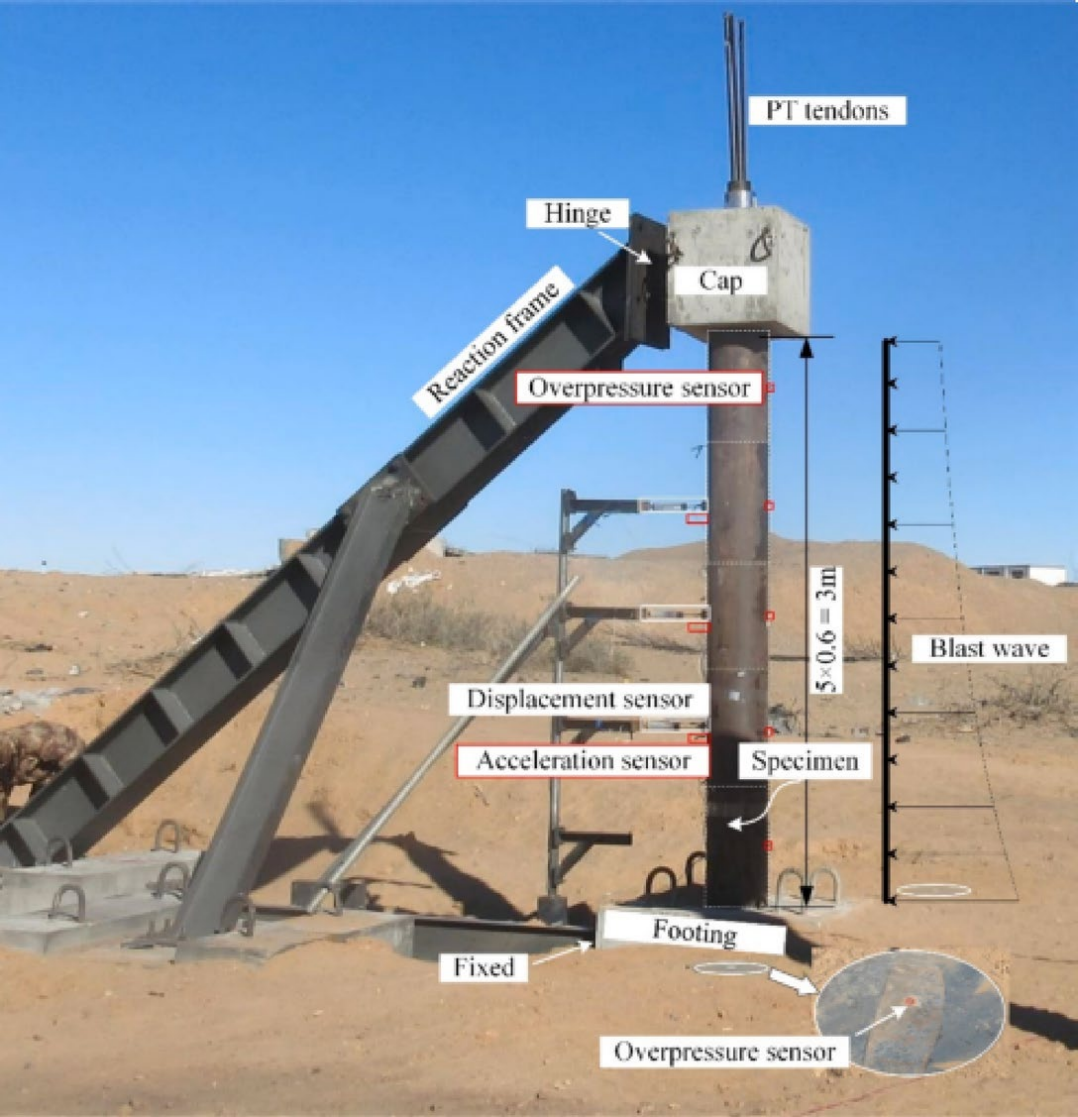
Overview of field blast test setup and detail of transducer arrangement^82^.

Xia et al.^82^ investigated experimentally and numerically the dynamic response of circular precast segmental concrete-filled double-skin steel tube (PS-CFDST) columns under close-in explosions. Three large specimens were used in the tests, each consisting of five segments with a height of 600 mm each. The outer tube dimensions were 325 × 6 mm, while the inner tube measured 159 × 6 mm. Two concrete blocks were placed at the top and bottom of each specimen. The top block had dimensions of 0.8 m × 0.8 m × 0.6 m (length, width, and height), while the bottom block measured 1.2 m × 1.0 m × 0.6 m. Two steel plates were placed between the column and the concrete blocks. Seven prestressed tendons were used to integrate the segments and concrete blocks. These tendons were placed inside the inner tube and were prestressed to a force of 500 kN, representing about 10% of the column’s ultimate axial load capacity. A schematic view of the specimen is shown in Fig. 18, and an overview of the test setup is provided in Fig. 19.

The first specimen had fixed-hinged boundary conditions and was subjected to 250 kg of TNT at a distance of 4.88 m. The other two specimens were subjected to 500 kg of TNT at a distance of 3.55 m, with one having fixed-hinged conditions and the other cantilevered. Xia et al. examined the failure patterns and fracture of post-tensioned (PT) tendons after the tests. They also developed numerical models using LS-DYNA, which were validated against the experimental results, to analyze the dynamic response, failure modes, and energy dissipation mechanisms. The study evaluated various modeling techniques for representing PT tendons and examined the influence of friction coefficients between segment interfaces. Xia et al. found that the dominant failure mode of PS-CFDST columns was shear failure, and that energy was dissipated through slippage between segments. The sleeve connection between CFDST segments significantly enhanced the column’s behavior under blast loading by dominating bending deformation, reducing joint slippage, and preventing fracture failure of the PT tendons.

## Conclusion

This research highlights the critical necessity of designing concrete-filled steel tube columns that can endure blast loads to preserve the structural integrity and safety of buildings and infrastructure. The paper summarizes and reviews currently available research on the response of Rc columns, CFST and CFDST columns to blast loading. The blast loading mechanisms, current design codes, different analysis techniques, and damage states of concrete columns under blast conditions were comprehensively reviewed. Recent research has investigated column behavior through analytical, numerical, and experimental approaches. While the analytical model can predict a column’s overall flexural and ductile blast response with acceptable accuracy, it fails to capture brittle damage modes like localized spalling and shear failure from close-in or contact explosions. Through the integration of experimental and numerical methods, researchers can achieve a thorough understanding of the dynamic response of columns to blast impacts. This paper reviews recent research that discusses the behavior of RC columns, as they are a traditional column shape, under blast loading. Furthermore, it examines recent studies that investigated the strengthening and retrofitting of RC columns. Moreover, by reviewing the effectiveness of various parameters on the blast response of CFST and CFDST columns—including tube axial load, thickness, section dimensions, concrete strength, and steel yield strength—key insights have been obtained. The results show that axial load at service levels, along with tube thickness, concrete strength, and steel yield strength, generally had a positive influence. Moreover, some previous studies have concluded that cross-sectional geometry has a significant influence, as it affects the damage pattern and mode of failure. Some previous research works have experimentally assessed column behavior through damage assessment, displacement, strain, and residual axial capacity measurements The most common research direction is to determine experimentally a column’s residual axial capacity, as this is the most effective method for assessing its ability to resist loads after a blast, especially from a contact explosion. These experimental tests are essential for validating and calibrating numerical model simulations. Several papers have numerically evaluated column blast response through damage indices, displacement, residual axial capacity, and energy dissipation analyses. Most of the numerical studies conducted with software such as LS-DYNA, AUTODYN, and ABAQUS, used nonlinear material models for concrete and steel. This nonlinearity effect was apparent through the deformation and rupture of the steel tube, as well as damage and erosion of the concrete particles inside it.

## Recommendations for future work

The behavior of reinforced concrete (RC) columns under contact, close-range, and far-range explosive loads has been extensively investigated in the existing literature. More recently, attention has shifted toward examining the performance of single and double concrete-filled columns subjected to contact and close-proximity blast events. However, several research gaps remain that warrant further investigation. To date, studies have predominantly focused on square and circular column cross-sections, with limited exploration of the response of rectangular concrete-filled steel tube (CFST) and concrete-filled double-skin steel tube (CFDST) columns under contact explosions. Given the critical influence of cross-sectional geometry on blast performance, this represents a significant gap in the literature. Also, a few of researches discus effect of fire’s temperature on performance of CFST and CFDST columns. One of the research gaps is using smart materials as filling material inside the tube (shape memory alloys, self-healing concrete, and geopolymer concrete) to enhance energy absorption.Retrofitting strategies are one of the research gaps that are recommended for future work, such as using fiber-reinforced polymer FRP-steel-concrete hybrid columns. Additionally, the behavior of CFST and CFDST columns under contact and near-field underwater explosions numerically and experimentally has not been addressed, despite its practical importance. This research field is considered one of the important fields to consider, as marine facilities such as petroleum platforms and marine wharves are often exposed to explosions, whether from accidents or terrorist attacks. The study of underwater explosions should include shock wave and gas bubble phenomena and compare the column’s behavior with air blast response. Additionally, the study could focus on several parameters such as geometry, standoff distance, tube thickness, concrete strength, and steel strength. One of the research gaps is the use of high-strength concrete, fiber-reinforced concrete, and geopolymer concrete as infill material inside tubes subjected to underwater explosions. These areas present valuable directions for future research.

## Data Availability

The datasets used and analyzed during the current study are available and published in journals as indexed in the reference list and can be provided by the corresponding author on reasonable request.
